# The Evolving Molecular Landscape and Actionable Alterations in Urologic Cancers

**DOI:** 10.3390/curroncol31110511

**Published:** 2024-11-06

**Authors:** Ryan Michael Antar, Christopher Fawaz, Diego Gonzalez, Vincent Eric Xu, Arthur Pierre Drouaud, Jason Krastein, Faozia Pio, Andeulazia Murdock, Kirolos Youssef, Stanislav Sobol, Michael J. Whalen

**Affiliations:** Department of Urology, The George Washington University School of Medicine & Health Sciences, Washington, DC 20052, USAmwhalen@mfa.gwu.edu (M.J.W.)

**Keywords:** urology, urologic cancers, genitourinary, prostate, bladder, renal cell carcinoma, testicular, penile, genetic mutations, molecular alterations

## Abstract

The genetic landscape of urologic cancers has evolved with the identification of actionable mutations that impact diagnosis, prognosis, and therapeutic strategies. This narrative review consolidates existing literature on genetic mutations across key urologic cancers, including bladder, renal, prostate, upper tract urothelial, testicular, and penile. The review highlights mutations in DNA damage repair genes, such as BRCA1/2 and PTEN, as well as pathway alterations like FGFR and PD-L1 overexpression. These mutations influence tumor behavior and therapeutic outcomes, emphasizing the need for precision oncology approaches. Molecular profiling, through tools like next-generation sequencing, has revolutionized patient care by enabling targeted treatment strategies, especially in cancers with distinct molecular subtypes such as luminal or basal bladder cancer and clear cell renal carcinoma. Emerging therapies, including FGFR inhibitors and immune checkpoint blockade, offer new treatment avenues, although resistance mechanisms remain a challenge. We also emphasize the importance of biomarker identification for personalized management, especially in metastatic settings where treatment intensification is often required. Future research is needed to further elucidate our understanding of the genetics affecting urologic cancers, which will help develop novel, individualized therapies to enhance oncologic outcomes.

## 1. Introduction

Cancer is a consequence of healthy cells undergoing cumulative mutations across singular or multiple genes, often over long periods of time. These series of mutations frequently confer a selective growth and survival advantage to the now abnormal cell, opening the door for further downstream mutations to foster uncontrolled growth [1]. These genetic mutations, therefore, underlie tumor behavior and have been linked to prognosis and response to therapy. Some genes are more potent in spurring abnormal cell growth and survival than others and can be referred to as “driver mutations”, implying their proliferative advantage. Other genes identified to be commonly mutated are the “passenger mutations”, which do not provide that same cell survival advantage [2]. Many such genes of each category have been identified since the dawn of the human genome project in 2004. Among the common groups of cancer mutations are those involving DNA damage repair (DDR) genes, DNA mismatch repair (MMR) genes, PTEN, FGFR, and RB1. DDR genes, such as BRCA1 and BRCA2, are tumor suppressor genes that maintain genomic stability by detecting and repairing DNA damage. Mutations in these genes lead to genomic instability and increased cancer risk. MMR genes and tumor suppressors correct DNA replication errors to prevent mutations; their deficiency results in microsatellite instability (MSI), associated with increased mutation rates and responsiveness to immune checkpoint inhibitors. PTEN is another tumor suppressor gene that regulates cell proliferation by antagonizing the PI3K/AKT pathway. Loss of PTEN function results in uncontrolled cell growth and is common in cancers like prostate cancer. FGFRs are proto-oncogenes involved in cell growth and differentiation; mutations or overexpression, particularly in FGFR2 and FGFR3, promote tumorigenesis and are targets for therapies in cancers such as bladder cancer. Lastly, RB1 is a tumor suppressor gene that controls cell cycle progression; mutations in RB1 lead to uncontrolled cell division and are found in various cancers, including bladder and prostate cancer.

The Cancer Genome Atlas (TCGA) project was a visionary, multi-institutional project that began in 2006 to explore the molecular landscape of 33 cancer types, including bladder, renal cell carcinoma (chromophobe, clear cell, and papillary), prostate, and testicular germ cell [3]. Beyond this landmark research collaboration, the modern era has witnessed exciting developments in the availability of whole exome sequencing through commercially available platforms that allow clinicians to incorporate this crucial information for clinical decision-making during real-time patient care. This information has moved from descriptive to decisive, truly from the bench to the bedside. However, more work is needed to identify and characterize the impact of these genetic changes in various domains such as cancer predisposition/heredity, cancer screening, impact/interplay of environmental factors, development of targeted therapeutics, precision-oncology tailored treatments and treatment intensification, and assessment of prognosis.

Genitourinary cancers maintain a significant burden of disease in the United States and around the world. According to the American Cancer Society, genitourinary cancers are estimated to make up 480,230 new cases and 68,600 deaths in 2024 [4]. Thus, genitourinary cancers are collectively responsible for nearly 23% of all new cancer diagnoses and 11% of cancer deaths in the U.S. [4]. With such far-reaching consequences of these cancers, extensive research has been conducted into their pathophysiological origins. Most current knowledge on genetic mutations in genitourinary cancers is based on hereditary syndromes, and a growing body of evidence identifies specific genetic associations within these cancers, which may enhance personalized diagnostic and treatment strategies. However, there is a notable lack of comprehensive reviews specifically on genetic mutations in urologic oncology. Therefore, this narrative review aims to comprehensively consolidate the existing literature on specific genetic mutations in urologic cancers, providing a more thorough understanding for future researchers and urologists.

### Methods

Using PubMed and Google Scholar, we performed a non-systematic review of articles. Articles selected were required to be original articles published in English. Information on clinical trials was collected from www.clinicaltrials.gov. Trials were selected based on the drug and urologic cancer of interest. We examined major urology and oncology journals and society guidelines.

## 2. Bladder Cancer

Bladder cancer (BCa) stands as the most prevalent malignancy within the urinary tract. It ranks among the most widespread cancers on a global scale, ranking as the seventh most common cancer among males and the eleventh among the general population [5]. In the United States, there are roughly 80,000 new cases of bladder cancer annually, and urothelial carcinoma accounts for 90% of these cases. The median age at diagnosis is typically around 70 years, with a notable male predominance [6].

Tobacco smoking is the most significant risk factor for bladder cancer, contributing to roughly half of all cases [5]. Occupational exposure to aromatic amines, aromatic polycyclic hydrocarbons, and chlorate hydrocarbons remains a significant risk factor. High arsenic levels in drinking water have also been associated with an elevated risk of bladder cancer. Factors such as fast acetylation or genetic predisposition may pose a risk [5].

About 80% of patients with BCa present with NMIBC, consisting of either noninvasive (pTa) or minimally invasive (pT1) BCa. Conservative approaches such as surveillance or intravesical Bacillus Calmette-Guérin (BCG) can be offered for patients with low to intermediate-risk BCa, respectively [7]. Generally, these patients exhibit a good prognosis [6]. For the remainder of patients with muscle-invasive BCa (MIBC), more aggressive treatment with multi-agent cisplatin-based chemotherapy and radical cystectomy is required [7]. Selecting optimal treatment regimens currently involves consideration of tumor histology, stage, and grade. Although molecular profiling has been reported by the TCGA and other research groups, the “actionability” of this information remains elusive. Indeed, a well-conducted previous attempt to ascertain neoadjuvant chemotherapy treatment response based on tumor molecular profile, the Southwest Oncology Group (SWOG) S1314 COXEN trial, was unsuccessful [8].

### Influence of Molecular Architecture on Treatment Response

Classically, bladder cancer has been divided histologically into urothelial and non-urothelial subtypes. However, with the increasing use of genetic sequencing technologies, our understanding of the molecular architecture of BCa has significantly expanded. BCa can be divided into molecular subtypes with distinct expression profiles, including luminal-papillary, luminal-infiltrated, luminal, basal-squamous, and neuronal [9]. Luminal subtypes are characterized by the upregulation of PPARG, GATA3, FOXA1, and uroplakin expression, representing urothelial differentiation. Additionally, luminal-type BCa can be subdivided by profiles with predominant FGFR3 expression, stromal infiltration signatures, or increased cell cycle markers [10]. Luminal BCa tumors are often noninvasive, but it has been proposed that some invasive tumors that show luminal expression signatures most likely evolve from the preexisting papillary disease and likely represent a progression of superficial papillary tumors [11].

While luminal BCa is less aggressive, these tumors have poorer responses to systemic chemotherapy compared to the more aggressive basal subgroups [12,13]. Tumors in the basal subgroup are categorized by the expression of markers such as p63, CD44, and keratins [14]. In contrast, BCa of the neuroendocrine subgroup has increased synaptophysin marker expression. In clinical practice, tumor expression profiling may direct guidance toward optimal systemic therapy options. Specific targeted therapies may have optimal responses depending on expression signatures. For example, favorable response with enfortumab-vedotin has been associated with increased nectin-4 response, which is more common in luminal-type BCa [15,16]. Additionally, unique molecular profiles have been postulated to correlate with favorable or poor response with intravesical BCG [17,18].

Microsatellite instability (MSI) has been associated with high mutation rates observed in various cancers (especially tobacco-related cancers). It has also been investigated for its role in predicting response with immune checkpoint inhibitor therapy. It has been estimated that MSI occurs in approximately 1% of MIBC and most often in patients with high-grade superficial disease [19,20]. Mismatch repair (MMR) gene deficiencies lead to replication errors and genetic instability, which typically manifests as somatic variations in the size of microsatellites, which present as short tandem repeat sequences in the genome. As demonstrated in the literature, approximately 10.3% of urothelial cancers exhibit an MMR deficiency [21]. Currently, both MSI and MMR have received approval as biomarkers for tumor-agnostic indications for pembrolizumab.

While immunotherapy has advanced the treatment of bladder cancer, targeting specific molecular pathways, such as the FGFR signaling pathway, offers an alternative therapeutic strategy. Erdafitinib, a novel FGFR inhibitor, has emerged as a promising treatment option for patients with FGFR2/FGFR3 alterations, particularly in BCG-unresponsive non-muscle-invasive and metastatic bladder cancer [22].

Erdafitinib (BALVERSA^®^), a pan-fibroblast growth factor receptor (FGFR) inhibitor, gained FDA approval in 2019 for the second-line treatment of locally advanced or metastatic urothelial carcinoma with FGFR2 or FGFR3 genetic alterations after platinum-based chemotherapy or immunotherapy failure [23]. FGFR alterations, particularly FGFR3 mutations and FGFR2/3 fusions, are prevalent in 50–80% of non-muscle-invasive bladder cancer and 20% of metastatic urothelial carcinoma cases [24,25]. These mutations often correlate with favorable progression-free survival but present challenges in recurrence-free survival due to immune evasion and poor response to immunotherapy. The mechanism of erdafitinib involves inhibiting the FGFR kinase pathway, preventing the phosphorylation of downstream proteins that mediate cell proliferation and survival, thereby reducing tumor growth. Erdafitinib’s efficacy in metastatic bladder cancer was demonstrated in the BCL-2001 trial, where patients with FGFR-altered urothelial carcinoma achieved a 40% overall response rate and a median PFS of 5.5 months [26].

Ongoing studies are exploring erdafitinib’s role in NMIBC. The THOR/BCL-2003 Phase II trial demonstrated a 100% complete response rate after three cycles for BCG-unresponsive patients with FGFR3 alterations [27]. Additionally, efforts are being made to develop intravesical delivery systems like TAR-210 to minimize systemic toxicities and improve local drug efficacy for NMIBC [28]. While erdafitinib has shown potential in treating both NMIBC and metastatic bladder cancer, further clinical trials are required to optimize its use with other therapies, such as immunotherapies, and better understand resistance mechanisms. Erdafitinib represents a pivotal advancement in the personalized treatment landscape of bladder cancer, particularly for patients harboring FGFR alterations. Table 1 summarizes key mutations in bladder cancer.

## 3. Renal Cell Carcinoma

Renal cell carcinoma (RCC) contributes to over 197,000 deaths annually worldwide and is the eighth most common cancer overall in the United States [31]. The average age at presentation is ≥60 years old, with a male predominance [32]. While there are known risk factors such as tobacco, obesity, hypertension, and CKD, the exact cause of the disease remains elusive [33].

The role of genetics in RCC has been known for decades based on well-described hereditary syndromes such as von Hippel–Lindau (VHL), tuberous sclerosis (TS), and Birt-Hogg-Dubé (BHD) syndrome. The identification and characterization of the molecular pathway of the VHL gene on chromosome 3 in both hereditary and sporadic clear cell RCC has also been widely reported, and this information has been used to develop the novel targeted molecular therapy belzutifan, a hypoxia-inducible factor (HIF)-2 alpha inhibitor.

The gold-standard treatment option for all histologic subtypes of RCC, when localized, is surgery with either partial or radical nephrectomy. In the metastatic setting, modern treatment relies on targeted therapies and immunotherapy, as most tumors are refractory to cytotoxic chemotherapy [34]. Targeted treatments include tyrosine kinase inhibitors (TKIs), which block angiogenic VEGF pathways and mTOR receptors [35]. The first-line treatment for metastatic RCC (mRCC) is typically combination therapy with immune checkpoint inhibitors (ICIs), such as nivolumab with ipilimumab, which block PD-1 and CTLA-4, respectively, or ICIs with TKIs, such as pembrolizumab with axitinib or avelumab with axitinib [34]. Specifically, nivolumab plus ipilimumab has been shown to extend the overall survival (OS) of mRCC compared to sunitinib alone (median not reached vs. 37.9 months; HR 0.71, *p* = 0.0003) [36,37].

### 3.1. Clear Cell Renal Cell Carcinoma

Clear cell renal cell carcinoma (ccRCC) accounts for 70% of all RCC cases [38]. This subtype is uniquely characterized by a germline mutation in the von Hippel–Lindau (VHL) gene (chromosome 3p25), hypermethylation of the gene, and complete loss of its resident chromosome 3p. Other genes on chromosome 3p are also often mutated in ccRCC, including BRCA-associated protein 1 (BAP1), polybromo-1 (PBRM1), and SETD2, which act as chromatin remodelers and tumor suppressor genes. Moreover, MMR and consequent MSI defects have also been observed [39,40].

Furthermore, immune system evasion through the expression of PD-L1 has been implicated in the development and progression of ccRCC, as in other cancer types. One meta-analysis of localized and advanced RCC demonstrated that up to 24% of clear cell renal cell carcinomas express PD-L1 compared to 10.9% of non-clear renal cell carcinomas (nccRCC) at the time of diagnosis [41]. A 2016 study showed that PD-L1 expression at the time of diagnosis was 12.6% in patients with metastatic ccRCC, in which 50% of patients received vascular endothelial growth factor (VEGF)-tyrosine kinase inhibitors (TKIs) therapy after surgery [42]. An increase in PD-L1 expression was associated with an increased mortality of over 50% [41]. There may be differential expression based on primary vs. metastatic site as well. A single institution analysis investigated n = 34 patients, comparing two tissue samples from different sites through immunofluorescence and automated quantitative analysis (AQUA). They found higher rates of expression of PD-L1 in metastatic tumors relative to the primary tumor [AQUA scores ranged from 5.1 to 32.7 (median: 15.5) for primary RCC tissue and from 8.1–51.7 for metastatic tissue (median: 21.7) [43]. The clinical implications here are that a single core biopsy in a patient with stage IV ccRCC may not provide a complete understanding of the tumor’s susceptibility to anti-PDL-1 drugs such as nivolumab. This makes PD-L1 challenging to use as a prognostic factor [43,44,45,46]. Metastatic tumors have been found to express PD-L1 at higher rates, which explains why they are more susceptible to anti-PDL-1 monoclonal antibodies [47]. However, their responsiveness to treatment is short-lived, possibly due to intra-tumor PD-L1 heterogeneous expression [44,47].

MMR mutations have also been described in clear cell renal cell carcinoma. These genes, including MLH1, MSH2, MSH6, and PMS2, function in concert to correct mutations that accumulate during DNA replication. Studies have shown that mutations in MMR vary among RCC subtypes. In ccRCC, there may be an almost complete loss of MLH1 expression (92% of patients), as the gene is located on chromosome 3p [39]. Studies have also demonstrated that mutated MSH2 in many sporadic RCC carcinomas can be observed, but the role MMR plays in the pathogenesis of any RCC remains unclear [45,48].

Moreover, SETD2 is a common mutation observed in ccRCC. The gene codes for histone lysine methyltransferase, which has been correlated with tumor aggressivity when its product, H3K36met3, is present, but SETD2 is lost [49]. H3K36met3 is an essential marker for the MMR protein complex. Thus, the loss of SETD2 correlates with a loss of function of MMR [50,51], leading to a loss of heterozygosity and MSI [48].

### 3.2. Papillary Renal Cell Carcinoma

Papillary renal cell carcinoma (pRCC) is the most common non-clear cell renal cell carcinoma (nccRCC), comprising 10–15% of all RCCs [52]. Although there are hereditary predispositions to pRCC, most occur sporadically. Traditionally, pRCC has been classed as either type I or type II, as differentiated by light microscopy. However, there is a growing movement toward a new classification schema based on these cancers’ mutational profiles. Type I pRCC is less aggressive than type II and genetically distinct. Type I pRCC is associated with mutations in MET, whereas type II is associated with SETD2 mutations, CDKN2A downregulation, and other abnormalities [53]. As in ccRCC, SETD2, PBRM1, and BAP1 mutations are also found in pRCC. However, unlike in ccRCC, the loss of chromosome 3p is not always seen [54]. pRCC has also been associated with mutations in well-studied pathways such as P53, mTOR, and Hippo [53,55].

Type I pRCC can cluster in families with a mutation in the proto-oncogene MET on chromosome 7. However, most cases occur through somatic mutations [56]. Type I pRCC can also arise from additional copies of chromosomes 7 and 17 [56]. Type II pRCC has been shown to have a hereditary component through the inheritance of a mutated fumarate hydratase gene involved in the tricarboxylic acid cycle. This gene, when mutated, leads to the development of hereditary leiomyomatosis and increases the risk of type II pRCC [57]. Mutations in the genes ARID2 (on chromosome 12) and CNOT1 (on chromosome 16) and gain of function mutations on chromosome 20 have also been associated with an increased risk of type II pRCC [56].

PD-L1 expression varies in each of these pRCC subtypes [58]. One study of localized and advanced RCC observed PD-L1 expression between type I pRCC and type II pRCC, noting that 22% of type I pRCC expressed PD-L1 versus 36% of type II [59]. Another study showed that PD-L1 expression was as high as 10% in pRCC tumor cells and was correlated with TNM stage but not patient age and tumor size. That same study found that 60% of patients with PD-L1+ tumor-infiltrating mononuclear cells had pRCC [60]. Additionally, PD-L1-positive tumors were associated with a significantly increased risk of mortality (HR = 6.41; *p* < 0.001) [60].

Papillary renal cell carcinoma has also been associated with MMR dysfunction. Unlike in ccRCC, where protein expression may be variable, MLH1 and MSH2 proteins are generally still expressed in pRCC, with studies indicating that these proteins are conserved in up to 25% of cases (n = 7/28; *p* < 0.001). A complete loss of MLH1 was observed in 68% of cases of pRCC (n = 18/28; *p* < 0.001), which is significantly below 92% (n = 80/87) in ccRCC [39]. Previous studies have shown that MLH1 protein expression can drop as low as 50%, but MSH2 expression remains intact [61]. Despite many tumors exhibiting a complete loss of MHL1, very few have shown high levels of microsatellite instability, making explicit connections between loss of MLH1 and MSH2 and MSI unclear. MSI correlates with metastatic nodal involvement (stage IV, N2) (n = 119; *p* = 0.008) [39]. Genetic variations between type I and type II pRCC remain unclear.

### 3.3. Chromophobe Renal Cell Carcinoma

Chromophobe renal cell carcinoma (cRCC) is the second most common non-clear cell renal cell carcinoma, comprising 5–10% of all RCC cases [62]. Chromophobe renal cell carcinoma is characterized by a loss in chromosomes 1, 2, 6, 10, 13, 17, and 21 [31]. Additionally, research has shown mitochondrial DNA mutations through upregulation of telomerase reverse transcriptase expression and common mutations in FLCN, TP53, and PTEN genes [34,63]. Mutations in FLCN lead to Birt-Hogg-Dubé syndrome, which progresses to cRCC in 34% of BHD (+) patients. TP53 has been found to be mutated in 32% (n = 21/66), while PTEN is mutated in 9% (n = 6/66) of cRCC cases [63].

Concerning PD-L1, its upregulation in cRCC has been observed at varying levels. Compared to ccRCC and pRCC, one study showed that cRCC tumors expressed PD-L1 at lower rates (5.6% of cases, n = 2/36) [60]. Tumor-infiltrating mononuclear cell (TIMC) was significantly higher at 36% (n = 13/36 cases). PD-L1 expression was associated with higher TNM stages and mortality (*p* < 0.05). Another study found that at the time of diagnosis, 13.6% (n = 81) of tumor cells were PD-L1+, and 30.9% of TIMC (n = 81) were PD-L1+ in metastatic cRCC. However, they did not find a statistically significant correlation between PD-L1 expression of the tumor cells and overall survival (91.9 and 76.4 months vs. 100 and 50%; *p* = 0.48) nor between PD-L1 expression on the TIMC and overall survival (90.5 and 72.2 months vs. 100 and 75%; *p* = 0.41) [43].

Disruptions in MMR protein synthesis can be seen in chromophobe renal cell carcinoma. The degree of dysfunction remains moderate, on par with pRCC, with full protein expression still seen in 33.3% (n = 12; *p* < 0.001) but complete absence in 58.3% (n = 12; *p* < 0.001). However, differences were seen in MSH2 expression, where cRCC maintained full protein expression in 41.7% (5/12 cases) and with only one complete loss of expression [39].

Microsatellite instability in cRCC has been documented. In one study analyzing 116 patients with various forms of non-clear cell renal cell carcinoma, it was found that two cases were considered to have high levels of MSI, one being cRCC [64]. The study further revealed that intermediate MSI was found in five other cases. Those with cRCC had the highest frequency of MSI (high and intermediate) at 24% [64]. Actionable targets across renal cell carcinomas may be found in Table 2.

## 4. Prostate Cancer

Prostate cancer (PCa) has become the most common non-skin malignancy in men in Western countries. It is the second most common cause of cancer deaths in American men, behind only lung cancer, with an estimated 299,010 new cases and 35,250 deaths in 2024 [65]. PCa’s increasing prevalence and its status as a leading cause of cancer-related deaths underscore the critical need for continued research and refined diagnostic and treatment strategies.

While traditional diagnostics such as prostate-specific antigen testing and biopsy have long been the cornerstone of prostate cancer detection and risk stratification, advances in molecular profiling have introduced gene expression classifiers that offer a deeper understanding of tumor biology. These tools, including Oncotype DX^®^, Decipher^®^ Genomic Classifier, and Prolaris^®^, to name a few, provide critical insights that complement conventional histopathology, enabling more personalized treatment strategies. These diagnostic advancements are instrumental in distinguishing patients who may benefit from active surveillance—those with indolent disease characterized by a low risk of progression—from those who may require definitive local treatment, such as surgery or radiation therapy, or even treatment intensification with androgen deprivation therapy. For instance, Oncotype DX^®^ provides a Genomic Prostate Score that aids in predicting the likelihood of adverse pathology and the potential for biochemical recurrence after surgery, thus guiding urologists’ decisions regarding the need for multi-modal treatment intensification. Moschovas et al. support the utility of the Oncotype DX’s GPS in predicting adverse pathological outcomes in low- to high-grade PCa patients undergoing radical prostatectomy [66]. The authors’ multivariable logistic regression analysis found that GPS was an independent predictor of extraprostatic extension (EPE) and seminal vesicle invasion (SVI), with odds ratios of 1.8 and 2.1, respectively, for every 20-point increase in GPS. Additionally, the percentage of cases with EPE and SVI increased consistently with higher GPS quartiles. These findings suggest that the Oncotype DX GPS can be instrumental in preoperative planning by identifying patients with a higher risk of these unfavorable pathological features, thereby improving patient counseling and informing surgical intervention. 

Similarly, the Decipher GC has been validated as a robust predictor of metastasis risk, which is crucial in determining the necessity of therapy post-prostatectomy. A recent study analyzed a large U.S. cohort using SEER program data, linking Decipher GC scores to treatment decisions and pathological outcomes. The study found that patients with high-risk Decipher scores were significantly more likely to experience adverse pathology at surgery, including pathological stage T3/4 and lymph node invasion (OR = 2.88, 95% CI = 1.35–6.17) [67]. Moreover, the use of postoperative radiotherapy was notably higher in patients with high Decipher scores (OR = 2.69, 95% CI = 1.89–3.84), suggesting that Decipher GC testing is instrumental in guiding post-surgical management to mitigate the risk of metastasis.

GC significantly influenced clinical decisions, particularly in stratifying patients for active surveillance or definitive treatment. Specifically, patients with lower Decipher scores were more likely to be managed conservatively with active surveillance (41% of low-risk GC patients). In comparison, those with higher scores were more frequently directed toward definitive treatments such as surgery or radiation [67]. A systematic review of 42 studies encompassing 30,407 patients demonstrated that the Decipher GC is independently prognostic for multiple oncologic outcomes, including adverse pathology, biochemical recurrence, distant metastasis, and cancer-specific survival. The review found that the GC significantly improves the discrimination over standard clinicopathologic models, with an area under the curve consistently enhanced when the GC is added [68]. Notably, the GC’s ability to change clinical management was highlighted, with studies showing that testing altered treatment decisions in the post-prostatectomy setting, where the number needed to test to change management ranged from 1.5 to 4. These findings underscore the utility of Decipher GC not only in guiding adjuvant therapy and refining surveillance strategies, ultimately contributing to more personalized and effective patient care.

Prolaris^®^, on the other hand, analyzes 31 cell-cycle progression genes (along with 15 housekeeper genes) to assess the 10-year prostate cancer-specific mortality risk, thereby informing decisions on whether to pursue immediate treatment or continue with surveillance [69]. These gene expression profiles collectively support a more individualized approach to cancer care, ensuring that intensified treatment is reserved for those who would benefit most while substantially minimizing overtreatment in patients with less aggressive disease.

The treatment of prostate cancer is complex and depends on stage, grade, and patient comorbidities; it often involves multi-modal therapies such as surgery, radiotherapy, androgen deprivation therapy, and even chemotherapy. Surgery and/or radiation are employed for curative intent in low- or intermediate-risk or locally advanced cases, ADT for advanced disease, and ablative therapies as primary or salvage options [70]. Immunotherapy is not widely used for treating PCa as the role of the immune system within prostate tumors is complex [71,72]. With targeted therapies emerging, new avenues exist for enhancing patient outcomes by addressing specific genetic alterations. Approximately 11% of prostate cancers globally show MSI or the loss of at least one MMR protein [73]. Furthermore, nearly 3–5% of prostate cancer cases are associated with a deficiency in MMR genes (MSH2, MSH6, PMS2, MLH1), resulting in hypermutation and MSI [74]. A growing body of literature suggests that PD-L1 expression is upregulated in prostate cancer tissues compared to normal tissues. However, the specific incidence has yet to be reported. PD-L1 mutations tend to be expressed in aggressive prostate cancer and are associated with poorer prognosis [75].

Mutations in MMR genes, namely MLH1 (chromosome 3), MSH2 (chromosome 2), MSH6 (chromosome 2), and PMS2 (chromosome 7), are associated with a 0.7–1.7% risk of hereditary prostate cancer and as high as 3.7% in sporadic PCa [76,77,78]. MMR mutations are relatively uncommon compared to other DNA damage repair genes [77]. In men with Lynch syndrome, these MMR gene mutations increase the risk of prostate cancer by two to three times, even when these individuals were not diagnosed with hereditary non-polyposis colorectal cancer [79]. While the precise role of these mutations in prostate cancer tumorigenesis requires further investigation into the precise mechanism, studies indicate that prostate tumors in patients with known MMR mutations often exhibit MMR deficiency, implying a role for germline alterations in predisposing individuals to prostate cancer [78,80].

Fang et al. examined the genomic landscape of mismatch repair-deficient (MMR-d) prostate cancers by analyzing data from 2664 primary and 1409 metastatic prostate tumors from the TCGA and GENIE databases [81]. MMR-d prostate cancers, which represent approximately 2.6% of primary and 4.3% of metastatic cases, were found to have distinct mutational profiles compared to mismatch repair-proficient (MMR-p) tumors. The study identified high frequencies of mutations in genes such as KMT2D (46.4% in primary and 33.3% in metastatic tumors) and JAK1 (31.9% in primary and 28.3% in metastatic tumors). The study also found that MMR-d tumors had significantly higher tumor mutational burden (TMB) than MMR-p tumors, with a median TMB of 0.82 mutations per megabase in MMR-d tumors versus 0.52 in MMR-p tumors. These findings underscore the potential for targeted therapies in MMR-d prostate cancers and highlight the importance of genomic profiling in identifying these tumors.

Moreover, Dasari et al. analyzed genomic data from 183 prostate cancer patients to explore the evolution of genetic alterations as prostate cancer progresses from primary to metastatic castrate-resistant prostate cancer (mCRPC) [82]. The study found that actionable genomic alterations, such as MSI-H and homologous recombination repair (HRR) gene mutations, were present in approximately 20% of the cases. Importantly, MSI-H was more common in metastatic disease (8.1%) compared to primary prostate cancers (1%). MSI-H was also associated with higher tumor mutational burden (TMB > 10 mutations/megabase) in 86% of MSI-H cases, highlighting its potential as a biomarker for immune checkpoint inhibitor therapy. These findings emphasize the need for comprehensive genomic profiling in advanced prostate cancer to guide personalized treatment strategies.

One large-scale genomic analysis of mCRPC patients identified mutations in DNA repair genes, including those involved in mismatch repair (MMR) and homologous recombination repair (HRR), prevalent in a subset of mCRPC cases [83]. Additionally, mutations of MSI-H and BRCA2 were associated with more aggressive disease phenotypes, underscoring the importance of genetic testing in guiding treatment decisions for mCRPC patients [84]. One study of 109 mCRPC patients showed that 50 had MMR alterations (seven pathogenic and 43 of unknown significance) [85]. In the subgroup with pathogenic MMR alterations, 42.9% had a Gleason score of ≥8, while in the group with MMR alterations of unknown significance, 62.8% had Gleason scores of ≥8. These data suggest a higher prevalence of aggressive disease (Gleason score ≥ 8) in patients with MMR alterations. Furthermore, the authors found that 3.7% of metastatic prostate cancers had deleterious alterations in MSH2, MSH6, or MLH1, which correlated with poorer responses to abiraterone and shorter progression-free survival.

Boiarsky et al. investigated the association between a panel-based mutational signature of MMR-d and the response to pembrolizumab in mCRPC [86]. The authors noted that mCRPC patients with a specific MMR-d mutational signature exhibited durable responses to pembrolizumab, with some patients achieving long-term disease control. Furthermore, alterations like EPCAM and MSH6 co-deletions were linked to enhanced immune checkpoint inhibition responses [86]. These findings suggest that identifying MMR-d signatures through genomic testing could help select patients most likely to benefit from pembrolizumab, highlighting the potential for personalized immunotherapy approaches in mCRPC. A case report described a patient with aggressive mismatch repair-deficient prostate cancer, characterized by a somatic co-deletion of EPCAM, MSH2, and MSH6, who achieved a complete radiographic response to pembrolizumab within three months, along with a significant drop in PSA levels. This co-deletion likely caused a profound deficiency in DNA mismatch repair, resulting in MSI-H and a heightened neoantigen load, enhancing the tumor’s immunogenicity and response to pembrolizumab. This case underscores the importance of genomic profiling in identifying patients with specific gene alterations who may benefit from immune checkpoint inhibitors, even in highly aggressive disease.

Moreover, tumors’ microsatellite instability and hypermutation are associated with a mismatch repair system deficiency (dMMR) resulting from mutational or epigenetic events [73]. This deficiency can increase mutation rates and is linked to chemoresistance and immunotherapy sensitivity [73]. Hypermutation is correlated with higher expression of tumor neoantigens, making tumors more recognizable by the immune system [73]. These aspects of MMR gene mutations and MSI further emphasize their relevance in the context of prostate cancer therapeutics [87,88]. To this end, clinical investigations have evaluated the efficacy of immune checkpoint inhibitors, particularly pembrolizumab, in this patient subpopulation. Pembrolizumab, an anti-PD-1 monoclonal antibody, was the first drug to receive tumor-agnostic approval from the FDA for high MSI or dMMR cancers, including prostate cancer, making it a key focus in the treatment of metastatic castration-resistant prostate cancer (mCRPC) with MSI-H status.

The seminal phase II KEYNOTE-199 trial assessed pembrolizumab in mCRPC patients who had progressed after docetaxel and endocrine therapies [89]. The study included 258 patients across three cohorts: PD-L1–positive, PD-L1–negative, and bone-predominant disease. The objective response rates (ORRs) were modest at 5% for PD-L1–positive and 3% for PD-L1–negative patients. However, the responses were durable, with a median duration of 10.6 months in PD-L1–negative patients, and the disease control rate in bone-predominant disease was 22%. Despite limited ORRs, the observed durability of responses suggests potential benefit in a subset of mCRPC patients, particularly those with bone-predominant disease, underscoring the need for further exploration of pembrolizumab in combination therapies.

Further supporting the role of pembrolizumab in MSI-H prostate cancer, a 5-year retrospective analysis conducted at the Mayo Clinic stratified 22 mCRPC patients by MSI-H and/or high tumor mutational burden and provided contemporary evidence of pembrolizumab’s efficacy [90]. The ORR among MSI-H patients was 75%, with a complete response rate of 27.3%. In contrast, patients with high tumor mutational burden but without MSI-H did not respond as favorably, underscoring the pivotal role of MSI-H in driving the effectiveness of pembrolizumab. These findings suggest that MSI-H status, rather than high tumor mutational burden alone, is a more reliable predictor of response to pembrolizumab in this patient population [90].

The 2020 NCI-MATCH trial (NCT02465060) was a landmark precision oncology study designed to evaluate targeted therapies across various cancer types based on specific genetic alterations rather than the primary tumor origin [91]. Of the 5954 screened patients, approximately 38% had actionable molecular alterations, and 18% were successfully assigned to treatment arms based on their genomic profile. Most notably, prostate cancer patients had a relatively high assignment rate of 23% compared to other common cancers. This trial showcases the feasibility of using advanced sequencing to guide patients. This trial also demonstrated the feasibility of using next-generation sequencing to identify patients who may benefit from targeted therapies, including those with MSI-H and MMR deficiency alterations. Many mutational targets in prostate cancer are summarized in Table 3.

## 5. Upper Tract Urothelial Cancer

Upper tract urothelial cancer (UTUC) constitutes 5–10% of all urothelial carcinomas, with an incidence rate of about two per 100,000 in Western countries, primarily affecting individuals over 70 years old [4]. UTUC can present unilaterally from the renal calyces to the ureteric orifices [92,93]. It is notably more aggressive than bladder cancer, evidenced by the two-thirds of cases that are invasive at diagnosis and the 22–47% recurrence rate after surgery [94,95].

Interestingly, a significant association exists between UTUC and Lynch syndrome, with approximately 9% of UTUC patients showing mutations in DNA mismatch repair genes [96,97]. MSH2 mutations are particularly prevalent in UTUC, contributing to a distinct genetic subtype of UTUC with marked microsatellite instability and a comparatively worse prognosis [98]. Despite these insights, the genetic characteristics of UTUC remain understudied, partly due to its rarity. More historically, this knowledge gap has continued to limit the development of optimal diagnostic and treatment strategies for UTUC. In fact, this cancer type was not included in the Cancer Genome Atlas Project [99]. However, next-generation sequencing has become a recent advancement to help address these notable gaps in our understanding.

The diagnosis of UTUC remains a challenge. Flexible ureteroscopy can be used for direct tumor visualization and biopsy. However, limited tissue yield from small-caliber biopsy equipment limits the use of routine molecular profiling prior to definitive surgical management. Urine cytology as an adjunctive tool has been proposed to improve diagnostic accuracy [100]. However, urine cytology has lower sensitivity for UTUC than bladder cancer and high false negative rates (50–90%), underscoring the need for more precise, non-invasive diagnostic methods [101,102].

For resectable UTUC, the primary treatment is radical nephroureterectomy (RNU) with bladder cuff resection, traditionally accompanied by perioperative platinum-based chemotherapy [94]. However, the development of targeted molecular therapies for UTUC has lagged behind bladder cancer, partly due to the limited representation of UTUC patients in critical bladder cancer clinical trials. However, recent trials (CHECKMATE-274 and EV302/KEYNOTE-A39) have included 20–30% of upper tract patients to investigate immune checkpoint inhibitors in their locally advanced unresectable and metastatic disease cohorts [103,104]. Since UTUC only comprises ~5% of urothelial carcinoma cases overall, this highlights the more aggressive nature of this disease.

Moreover, fibroblast growth factor receptor 3 (FGFR3) alterations are prevalent in UTUC, occurring in 35–56% of cases and even more so in sporadic UTUC (40–80%) [29,105,106,107,108,109]. Tumors with FGFR3 mutations often have reduced CD8 T-cell gene signatures, indicating FGFR3’s role in driving a T-cell-depleted immune environment [108]. Moreover, mutations in chromatin remodeling genes (KMT2D/KDM6A) and tumor suppressor genes (TP53) are observed in 35–56% and 18–26% of UTUC cases, respectively, with FGFR3 mutations correlating with a better prognosis. In contrast, TP53 mutations indicate a more aggressive disease [105,110]. The FDA-approved pan-FGFR inhibitor, erdafitinib, has shown promise, particularly given the high incidence of FGFR3 mutations in UTUC. In a phase II trial involving 99 patients, of whom 23 had UTUC, the response rate to erdafitinib was 40%, with UTUC patients showing a 43% (95% CI 23–64) response rate [26].

Furthermore, investigations into PD-L1 mutations, found in up to 20–25% of UTUC tumors and associated with worse survival, have highlighted this as a potential target for immunotherapy [111,112], as well as targeting HER2 with trastuzumab [113]. Pembrolizumab and atezolizumab, approved for cisplatin-unfit PD-L1+ patients with unresectable or metastatic UTUC, have shown efficacy. The KEYNOTE-052 trial demonstrated a 24% overall objective response rate with pembrolizumab (38% in patients with a PD-L1 expression over 10%) and a six-month overall survival rate of 67% (95% CI: 62–73%). More notably, UTUC patients constituted 19% of the 370 trial participants, and their response rates (22%) were comparable to those with bladder cancer (28%) [114]. A summary of mutations and targets in upper tract urothelial cancer are found in Table 4.

## 6. Penile Cancer

Penile cancer, while rare and representing less than 1% of male cancers in the United States, is considerably more prevalent in less developed regions of the world [115]. Specifically in these areas, it has posed a substantial public health concern, accounting for 10–20% of male malignancies [116]. Despite a declining incidence of penile cancer over the past three decades, approximately 2100 new cases and 500 deaths are reported annually in the United States [117]. Understanding the risk factors associated with penile cancer is critical in reducing its occurrence and promoting prompt detection. Notable risk factors include human papillomavirus (HPV) infection, tobacco usage, uncircumcised status and the presence of phimosis, and immunosuppressive conditions (i.e., HIV infection) [118,119,120]. Addressing these factors effectively can reduce the risk of penile cancer development. Moreover, the early detection of penile cancer and inguinal lymph node metastasis remains the most effective strategy for limiting the associated morbidity and mortality [121].

The diagnosis of penile cancer generally requires histological analysis through biopsies and subsequent staging to ascertain the extent of the disease, which guides the appropriate treatment. Standard interventions include a combination of chemotherapy, radiation therapy, micrographic surgery, glansectomy, and, in severe cases, penectomy [121]. Notably, some penile cancer cases exhibit resistance to conventional chemotherapeutic agents such as cisplatin [122], prompting the exploration of alternative treatment methods (i.e., targeting HER2) [123].

To date, there has been no evidence of MSI or MMR deficiency in squamous cell carcinoma (SCC) of the penis. A study by Stoehr et al., which involved an analysis of MSI status and MMR protein expression in 105 penile SCC samples (median stage of T1a, median grade 2, with multiple histologies), found that these genetic alterations are likely not characteristic of penile SCC [124]. Consequently, these findings suggest that MSI and loss of MMR genes may not serve as effective biomarkers for PD-1/PD-L1 blockade therapy in treating this specific cancer subtype.

However, PD-L1 expression has been documented in many series. In a large cohort of 222 men undergoing treatment for penile SCC, the differential expression of PD-L1 across various stages was evident when assessed with two distinct antibody clones, SP142 and 28.8 [125]. Specifically, PD-L1 expression in tumor cells using the SP142 clone was observed in 12.50%, 25.00%, 31.25%, and 31.25% of cases for carcinoma in situ, pT1, pT2, and pT3 stages, respectively. In contrast, when using the 28.8 clones, the incidence was 4.48%, 31.34%, 38.81%, and 25.37% for the corresponding stages.

These findings underscore a stage-dependent variability in PD-L1 expression, with both antibody clones showing a peak in expression at the pT2 stage. The relatively higher percentages detected with clone 28.8, particularly in the pT1 and pT2 stages, suggest that the choice of diagnostic antibody could significantly impact the perceived prevalence of PD-L1 expression and, by extension, therapeutic decisions. Given that PD-L1 is a pivotal biomarker for immunotherapeutic strategies targeting the PD-1/PD-L1 axis, the observed expression patterns may influence the selection of candidates for such treatments, highlighting the importance of stage-specific considerations in penile SCC management.

PD-L1 positivity in tumor cells, as detected by either SP142 or 28.8 antibody clones, was significantly linked with shorter cancer-specific survival. Specifically, median survival for patients with PD-L1-positive tumors was 1.5 years when detected with SP142 and 1.94 years with the 28.8 antibody. This is in comparison to 3.12 years and 4.35 years for PD-L1-negative patients, respectively. Notably, the prognostic significance of tumor-cell PD-L1 expression remained even after adjusting for histological grade, TNM stage, and high-risk HPV status (HR = 4.37 [1.04–18.32]). Furthermore, a study found that PD-L1 expression was strongly correlated with regional node metastasis [126].

Reliable prediction of regional nodal involvement through gene expression profiling would be critical to informing the role of adjuvant inguinal lymphadenectomy vs. surveillance, given the morbidity of surgery. For instance, 18 F-FDG PET imaging is reliable for assessing metastatic disease in patients with palpable inguinal lymph nodes, showing high sensitivity rates. However, challenges remain in detecting clinically occult (i.e., non-palpable) metastases, where their sensitivity drops. Advancements in diagnostic methods are continually being sought to improve the detection and staging of this disease.

The presence of PD-L1 expression may inform treatment choices, advocating for tailored therapies. Of note, most of the immune checkpoint inhibitor studies were retrospective studies. The PULSE trial investigated the effects of avelumab, an anti-PD-L1 therapy, as a maintenance treatment after platinum-based chemotherapy in patients with locally advanced disease [127]. The median duration of avelumab maintenance was 3.7 months, mainly due to disease progression in most patients. Another study, the ORPHEUS trial, assessed the effectiveness of retifanlimab, a PD-1 inhibitor, in cases with diverse disease courses, most of which had previous surgical resection with lymph node dissection. The objective response rate was 16.7% (95% CI 5.8–39.2%), with three partial responses, and the median duration of response and therapeutic range were 3.3 months and 1.9 months, respectively. The median progression-free survival was 2.0 months (95% CI 1.6–3.3), and overall survival was two months (95% CI 3.0–9.8) [128]. Additionally, a separate study evaluated pembrolizumab monotherapy in 127 patients with advanced rare diseases, including three patients with PSCC. One patient had a partial response, while the other two experienced disease progression during treatment. Developing novel therapeutic approaches includes HPV-directed therapy, anti-EGFR agents, and anti-PD-L1 [129,130,131].

Understanding resistance mechanisms is vital, especially given the common occurrence of resistance to first-line therapies like cisplatin. Tumors that exhibit resistance due to increased homologous recombination deficiency may also show decreased sensitivity to drugs like PARP inhibitors. Furthermore, alternatives such as HER2 targeting in cisplatin-resistant tumors is an ongoing and exciting area of investigation, given that HER2 over-expression has been linked to poor survival in penile squamous cell carcinoma with an immunohistochemical expression rate of approximately 47.7% [132]. In subsequent evaluation, Kaplan–Meier survival curves demonstrated that as HER2 expression scores increased, the OS and DFS of patients worsened. Furthermore, growing evidence has shown that HER2 overexpression can even induce chemo-resistance, as seen in gastric cancers [133]. This suggests that HER2 targets remain viable for cisplatin-resistant lesions [123].

Currently, clinical trials are ongoing for targeted therapies, including those directed at HER2, creating an avenue for advancements in future penile cancer care. As we gain a deeper understanding of these genetic characteristics of this patient population, more effective therapies may emerge and inform the treatment of other genitourinary cancers. Various mutations found in penile cancer are summarized in Table 5.

## 7. Testicular Cancer

Although they comprise only 1% to 2% of all adult male neoplasms, testicular germ cell tumors (GCTs), representing about 95% of testicular cancers, are the most common malignant neoplasms in young men of European ancestry [134,135,136,137]. About 9760 new cases of testicular cancer will be diagnosed in the United States in 2024, making it the most common cancer in men aged 15–34 [138]. The carcinogenesis of GCTs is poorly understood. Transformed primordial germ cells are thought to lay dormant until after puberty, stimulated by increased testosterone levels. Except for spermatocytic tumors, postpubertal testicular and extragonadal germ cell tumors are consistently found to contain more copies of the short arm of chromosome 12 [139]. GCT cure rates have increased with the advent of multi-modal therapy with cisplatin-based chemotherapy and surgery [134], and the long-term survival for men with metastatic GCT is over 70% [140].

GCTs are a complex group of tumors with heterogeneous histology and can be categorized as seminoma and nonseminoma [136]. Nonseminoma can be further divided into embryonal carcinoma, teratoma, yolk sac tumor, and choriocarcinoma, with teratoma being the most chemo-resistant GCT subtype [136]. European ancestry, cryptorchidism, a personal history of testicular cancer, a family history of testicular cancer, and germ cell neoplasia in situ are well-established risk factors for testicular cancer [141]. Other risk factors include sub- and infertility, environmental and/or lifestyle factors, and genetic susceptibility [142,143,144,145].

Recent research has explored the connections between malignant tumor biology and immune mechanisms. Immune checkpoints are frequently expressed in primary and metastatic GCTs, and variation in these markers’ expression levels is associated with a worse prognosis. One of the targets of immunotherapy in GCTs is PDL-1, which is a pro-tumorigenic factor when bound to its receptor [146]. In a sample of N = 158 GCTs, it was reported that 85.5% of the tumors expressed PD-L1 and that patients with lower or absent PD-L1 positive immune cells exhibit significantly worse relapse-free survival (HR = 4.481, *p* = 0.013) [136]. Additionally, PD-L1 immunoexpression was observed in 17/17 (100%) immune cells and 8/17 (47.1%) tumor cells of metastatic samples [136].

Tumor-associated macrophages (TAMs) have also been implicated in immune suppression and tumor aggressiveness. A 2020 retrospective study of N = 77 orchiectomy specimens found that GCTs, except choriocarcinoma, primarily expressed PD-L1 on TAMs and that seminomas had increased intratumoral PD-L1+ TAMs compared with nonmetastatic nonseminomatous germ cell tumors (*p* < 0.05) [134]. PD-L1 positive tumor cells were significantly more frequent in choriocarcinoma [134]. A 2016 translational study of N = 140 patients with GCTs reported that, in comparison to GCT patients with lower expression of PD-L1 (80%), patients with high infiltration of PD-L1 positive tumor-infiltrating lymphocytes (TILs) (20%) were found to have significantly better progression-free survival (HR = 0.40 [0.16–1.01], *p* = 0.008) and overall survival (HR = 0.43 [0.15–1.23], *p* = 0.040) [147]. Compared to other GCT subtypes, seminoma (n = 46, *p* < 0.0001) and embryonal carcinoma (n = 72, *p* < 0.0001) were associated with a higher frequency of PD-L1 positive TILs [147]. Treatment with pembrolizumab in platinum-refractory GCTs was evaluated in a phase II trial of n = 12 patients, demonstrating that pembrolizumab was well tolerated but lacked meaningful clinical efficacy in this setting [148]. No partial or complete responses were observed, although two patients achieved radiographic stable disease. However, these patients experienced continued rising AFP levels despite radiographic stability.

Mismatch repair deficient cancers tend to react favorably to treatment with immune checkpoint inhibitors. It has been reported that MMR deficiency and subsequent genomic instability trigger the formation of neoantigens and immunogenicity and often indicate a favorable response to anti-PD-L1 agents [136]. To screen for tumors with MMR deficiency, IHC analyzes the protein expression of MLH1, MSH2, MSH6, and PMS2 on tissue microarray. There are notable differences in MMR protein expression between the different GCT subtypes. For instance, low MSH2 scores are less common in seminomas, while low MLH1 and MSH2 scores are more common in teratoma and choriocarcinoma [149]. Earlier studies observed MSI and MMR deficiency in a subset of testicular GCTs. Still, more contemporary studies have shown that GCTs typically retain MMR expression when analyzed by IHC. According to Dum et al., of n = 536 GCT cases, 481 (89.7%) had unequivocally intact MMR protein expression [150]. One or more IHC stains were equivocal in 55 GCTs, and there was a lack of detectable MMR protein in both tumor and stromal cells [150]. They concluded that MMR deficiency had to have happened early in development, most likely around the transition from non-invasive to invasive seminoma. Additionally, they suggested that it has no bearing on lymphocyte influx in seminoma.

Cytotoxic T-lymphocyte-associated protein 4 (CTLA-4), a protein receptor that functions as an immune checkpoint and mediates interaction between antigen-presenting cells and T cells in the lymph node, is another critical marker. CTLA-4 immunoexpression can be found in immune cells [151]. Lobo et al. found that of N = 162 GCTs, 96.3% exhibited CTLA-4 positivity in immune cells [136]. Higher expressions were associated with favorable prognostic characteristics, including lower pT and N stage and less lymphovascular invasion. To date, the efficacy of PD-L1 and CTLA-4 checkpoint inhibitors in treating cisplatin-refractory germ cell tumors has not been established outside of clinical trials. However, better clinical outcomes may come from the potential success of other immunotherapy checkpoint inhibitors besides PD-L1 and CTLA-4 inhibitors. TILs and tumor mutation burden may be potential alternative predictors [151], but more research is required to understand their utility in clinical practice. Table 6 summarizes key mutations in testicular cancers.

## 8. Conclusions

This narrative review aimed to provide a comprehensive state-of-the-art view into the incidence and therapeutic landscape of key genetic mutations in urologic oncology. Identifying and characterizing these mutations are pivotal steps in advancing predictive models and developing targeted therapeutic strategies. Continued research and collaborative efforts across multiple disciplines are essential to further elucidate the genetic landscapes of these cancers, ultimately enhancing patient care and outcomes in urologic oncology.

## Figures and Tables

**Table 1:** Bladder cancer.

Cancer Type	Mutation	Clinical Relevance	Author	Journal	Institution	Year	Cohort Size	Disease State	Drug	Drug Mechanism	Primary Outcome	Major Findings
Bladder Cancer	FGFR3	Common in luminal BCa; targeted by erdafitinib	Loriot et al. [29]	New England Journal of Medicine	Gustave Roussy Department of Cancer Medicine	2023	136	Metastatic	Erdafitinib	pan-fibroblast growth factor receptor (FGFR) inhibitor	Response Rate	12.1 months OS, 5.6 months PFS. Erdafitinib therapy resulted in significantly longer overall survival than chemotherapy among patients with metastatic urothelial carcinoma and FGFR alterations after previous anti–PD-1 or anti–PD-L1 treatment.
Bladder Cancer	MSI and MMR	Associated with response to immune checkpoint inhibitors	Balar et al. [30]	Lancet Oncology	54 sites in 14 countries, led by Perlmutter Cancer Center at NYU Langone Health	2021	101	High-risk NMIBC unresponse to BCG	Pembrolizumab	Monoclonal antibody against PD-1 receptor	Complete Response	41% had complete response at 3 months.
Bladder Cancer	NECTIN4	Nectin-4 is a cell adhesion molecule implicated in many cellular processes	Koshkin et al. [16]	Cancer	16 institutions	2022	304	Locally advanced or metastatic	enfortumab vedotin	antibody-drug conjugate against Nectin-4 protein	Response Rate	52% observed response rate. Median PFS and OS of 6.8 and 14.4 months, respectively.
Bladder Cancer	ERBB2	Target for trastuzumab	Seiler et al. [13]	European Urology	University of British Columbia	2017	343	Metastatic	Trastuzumab	targets the overexpressed ERBB2 (HER2) receptor, inhibiting tumor growth and promoting immune-mediated cancer cell destruction.	OS, PFS	OS: 14.2 months, PFS: 6.8 months

**Table 2:** Renal cell carcinoma.

Cancer Type	Mutation	Clinical Relevance	Author	Journal	Institution	Year	Cohort Size	Disease State	Drug	Drug Mechanism	Primary Outcome	Major Findings
Renal Cell Carcinoma	VHL	Target for HIF-2 alpha inhibitors	Fallah et al. [40]	Clinical Cancer Research	FDA approved (multiple sites)	2022	61	RCC associated with VHL disease	Belzutifan	HIF-2α inhibitor	Overall response rate	Objective response rate of 49%. Led to FDA approval of drug.
Clear Cell RCC	PD-L1	Upregulated in metastatic sites; target for ICIs	Motzer et al. [37]	Lancet Oncology	Memorial Sloan Kettering Cancer Center	2022	323	Advanced/metastatic clear cell RCC	Nivolumab + cabozatinib	PD-1 inhibitor and Tyrosine Kinase inhibitor	Progression free survival	Superior OS and progression free survival with novolumab plus cabozantinib versus sunitinib
Clear Cell RCC	PD-L1	Upregulated in metastatic sites; target for ICIs	Au et al. [46]	Cancer Cell	The Francis Crick Institute	2021	15	Intermediate or poor risk	Nivolumab	PD-1 inhibitor	Response Rate	5 out of 15 patients responded to Nivoloumab. Nivolumab drives both maintenance and replacement of previously expanded T cell clones, but only maintenance correlates with response.
Papillary RCC	MET	Mutation common in type I pRCC	Choueiri et al. [58]	Journal of Clinical Oncology	Dana-Farber Cancer Institute (DFCI)	2013	74	Advanced PRCC (locally advanced, bilateral multifocal, or metastatic)	Foretinib	MET/VEGFR2 inhibitor	Response Rate	ORR of 13.5%, progression-free survival of 9.3 months viewed favorably in comparison to VEGFR and mTOR inhibitors.
Renal Cell Carcinoma	TSC1, TSC2, MTOR	Dysregulated tumor suppressor gene as well as a component of the mTOR signaling pathway	Kwiatkowski et al. [55]	Clinical Cancer Research	Dana-Farber Cancer Institute	2016	79	Metastatic	Rapalogs	bind to FKBP12 to inhibit mTORC1 kinase activity	Partial Response Rate	Mutations in MTOR, TSC1, or TSC2 were more common in responders, 12 (28%) of 43, than non-responders, 4 (11%) of 36 (*p* = 0.06).

**Table 3:** Prostate Cancer.

Cancer Type	Mutation	Clinical Relevance	Author	Journal	Institution	Year	Cohort Size	Disease State	Drug	Drug Mechanism	Primary Outcome	Major Findings
Prostate Cancer	MSI/MMR	Associated with hypermutation and immunotherapy sensitivity	Le et al. [80]	New England Journal of Medicine	Sidney Kimmel Comprehensive Cancer Center at Johns Hopkins	2015	41	Progressive metastatic carcinoma	Pembrolizumab	Monoclonal antibody against PD-1 receptor	Response Rate and PFS	MSI/MMR deficiency linked to better response
Prostate Cancer	PD-L1	Upregulated in aggressive PCa	Xu et al. [75]	Molecular Therapy	the First Affiliated Hospital of Wenzhou Medical University	2021	300	Advanced	N/A	N/A	Biomarker	Higher PD-L1 expression linked to poor prognosis
Prostate Cancer	BRCA	Role in DNA repair; target for PARP inhibitors	He et al. [74]	Signal Transduction and Targeted Therapy	Shanghai Key Laboratory of Regulatory Biology	2022	400	Metastatic	Olaparib	poly (ADP-ribose) polymerase (PARP) inhibitor	Survival Rate	BRCA mutations linked to better response to PARP inhibitors. Olaparib approved by FDA in 2020 for treatment of mCRPC with deficient HR genes.
Prostate Cancer	AR-V7	Resistance mechanism to AR-targeted therapies	Scher et al. [72]	European Urology	Memorial Sloan Kettering Cancer Center	2017	161	Metastatic CRPC	Abiraterone, Enzalutamide	Androgen inhibitors	OS	AR-V7 positive associated with longer survival time on chemotherapy
Prostate Cancer	BRCA2	BRCA2 is the most commonly altered DDR gene in prostate cancer	Pomerantz et al. [84]	Cancer	Dana Farber Cancer Institute	2017	141	Metastatic CRPC	carboplatin/docetaxel	Platinum-based chemotherapy	Response Rate	BRCA2-associated CRPC is associated with a higher likelihood of response to carboplatin-based chemotherapy than non-BRCA2-associated prostate cancer.
Prostate Cancer	Various DNA- damage response (DDR) genes (BRCA1/2, ATM, CHEK2, FANCA, PALB2, HDAC2)	These DNA repair genes are found to be associated with PARP- inhibitor sensitivity	Mateo et al. [87]	New England Journal of Medicine	The Royal Marsden NHS Foundation Trust	2015	50	Metastatic CRPC	Olaparib	poly (ADP-ribose) polymerase (PARP) inhibitor	Response Rate	Treatment with the PARP inhibitor olaparib in patients whose prostate cancers were no longer responding to standard treatments and who had defects in DNA-repair genes led to a high response rate.
Prostate Cancer	Various DNA- damage response (DDR) genes (BRCA1, BRCA2, PALB2, ATM, ATR, CHEK2, FANCA, RAD51C, NBN, MLH1, MRE11A, CDK12)	These DNA repair genes are found to be associated with PARP- inhibitor sensitivity	Agarwal et al. [88]	The Lancet	223 Hospitals in 23 countries	2023	805	Metastatic CRPC	Talazoparib + Enzalutamide	poly (ADP-ribose) polymerase (PARP) inhibitor and androgen receptor inhibitor	Radiographic PFS	Talazoparib plus enzalutamide resulted in clinically meaningful and statistically significant improvement in rPFS versus standard of care enzalutamide as first-line treatment for patients with mCRPC.

**Table 4:** Upper tract urothelial cancer.

Cancer Type	Mutation	Clinical Relevance	Author	Journal	Institution	Year	Cohort Size	Disease State	Drug	Drug Mechanism	Primary Outcome	Major Findings
Upper Tract Urothelial Cancer	FGFR3	Common mutation; target for erdafitinib	Loriot et al. [26]	New England Journal of Medicine	Gustave Roussy Department of Cancer Medicine	2023	266	Metastatic	Erdafitinib	FGFR kinase inhibitor	Response Rate	43% response rate in UTUC patients.
Urothelial Cancer (upper tract, bladder, and urethra)	PD-L1	Upregulated; target for immunotherapy	Balar et al. [114]	Lancet	Perlmutter Cancer Center, New York University Langone Medical Center	2017	123	Unresectable/metastatic	Atezolizumab	Monoclonal antibody against PD-1 receptor	Response Rate	24% response rate with PD-L1 expression over 10%. Patients with PD-1/PD-L1- positive tumors should be offered a checkpoint inhibitor.
Upper Tract Urothelial Cancer	MSI, MMR (MSH2)	Linked to Lynch syndrome and MSI	Rouprêt et al. [98]	Urology	Tenon Hospital	2005	80	invasive upper urinary tract transitional cell carcinoma	N/A	N/A	Biomarker	High MSI is an independent factor linked to better prognosis in invasive UUT-TCC
Upper Tract Urothelial Cancer	ERBB2 (HER2)	Target for trastuzumab	Yorozu et al. [113]	Clinical Genitourinary Cancer	The Jikei University School of Medicine	2020	148	Molecular subtypes of urothelial carcinoma of the renal pelvis and ureter	N/A	N/A	HER2 protein overexpression/gene amplification and overall survival	HER2 overexpressed in 14% of UCRP cases, significantly associated with luminal subtype. OS of patients with HER2-positive UCRPU significantly shorter than HER2-negative tumors.

**Table 5:** Penile cancer.

Cancer Type	Mutation	Clinical Relevance	Author	Cohort Size	Disease State	Drug	Primary Outcome	Major Findings
Penile Cancer	HER2	Overexpression linked to poor prognosis; potential target	Tan et al. [123]	50	Chemoresistant	Anti-HER2	Response Rate	HER2 targeting viable for cisplatin-resistant tumors
Penile Cancer	PD-L1	biomarkers for PD-1/PD-L1 blockade therapy in penile carcinoma	Stoehr et al. [124]	75	Various	N/A	Biomarker	MSI and defects in MMR protein expression are not regular features of penile SCC and might not act as biomarkers for PD-1/PD-L1 blockade therapy in penile carcinoma.
Penile Cancer	PD-L1	clinical significance of PD-L1 expression in penile SqCC	Udager et al. [126]	37	Various	N/A	Biomarker	PD-L1 associated with high-risk clinicopathologic features and poor clinical outcome
Penile Cancer	PD-L1	anti PD-L1 immunotherapy in maintenance among patients with locally advanced or mSCPC	Gassian et al. [127]	32	advanced/metastatic PSCC	avelumab	PFS	After the first line, the prognosis remains poor with no consensus on a second line systemic treatment in locally advanced or mSCPC
Penile Cancer	PD-L1	anti PD-L1 immunotherapy in maintenance among patients with locally advanced or mSCPC	García Del Muro et al. [128]	18	advanced/metastatic PSCC	Retifanlimab	PFS	Single-agent retifanlimab exhibited signals of clinical activity in advanced/metastatic PSCC, with no new safety signals
Penile Cancer	PD-L1	anti PD-L1 immunotherapy in mSCPC	Baweja et al. [131]	1	Metastatic	ipilimumab and nivolumab	PFS	Refractory penile cancer with dramatic clinical response to combined checkpoint inhibition with ipilimumab and nivolumab.

**Table 6:** Testicular cancer.

Cancer Type	Mutation	Clinical Relevance	Author	Journal	Institution	Year	Cohort Size	Disease State	Drug	Drug Mechanism	N/A	Major Findings
Testicular Cancer	PD-L1	Upregulated in metastatic samples	Lobo et al. [136]	Cancers	Portuguese Oncology Institute of Porto	2019	162	Metastatic	N/A	N/A	Biomarker	85.5% PD-L1 expression linked to worse prognosis
Testicular Cancer	MMR	Rare; potential marker for immunotherapy	Dum et al. [150]	Translational Andrology and Urology	University Medical Center Hamburg-Eppendorf	2021	536	Various stages	N/A	N/A	Biomarker	89.7% had intact MMR protein expression
Testicular Cancer	CTLA-4	Immune checkpoint; target for immunotherapy	Lobo et al. [136]	Cancers	Portuguese Oncology Institute of Porto	2019	162	Various stages	N/A	N/A	Biomarker	High CTLA-4 expression linked to better prognosis
Testicular Cancer	PD-L1	Differences in immune microenvironment	Sadigh et al. [134]	American Journal of Clinical Pathology	Hospital of the University of Pennsylvania	2020	77	Various	N/A	N/A	Biomarker	Robust PD-L1+ TAMs are significantly expanded in seminomas compared with NSGCTs
